# Readability of Online Spanish Materials for Breast Reconstruction Using Deep Inferior Epigastric Perforator Flaps

**DOI:** 10.7759/cureus.64616

**Published:** 2024-07-15

**Authors:** Cameron Gerhold, Timothy E Nehila, Virginia Bailey, Bilal Koussayer, Mohammad Tahseen Alkaelani, Nicole K Le, Mahmood Al Bayati, Kristen Whalen, D’Arcy Wainwright, Deniz Dayicioglu

**Affiliations:** 1 Orthopedic Surgery, Florida State University College of Medicine, Tallahassee, USA; 2 Surgery, University of South Florida Health Morsani College of Medicine, Tampa, USA; 3 Plastic Surgery, Florida State University College of Medicine, Tallahassee, USA; 4 Clinical Sciences, Florida State University College of Medicine, Tallahassee, USA; 5 Plastic Surgery, University of South Florida, Tampa, USA

**Keywords:** autologous breast reconstruction, plastic and reconstructive surgery, spanish health education, deep inferior epigastric artery perforator flap, procedural education, readability

## Abstract

Background

The internet has become an increasingly popular tool for patients to find information pertaining to medical procedures. Although the information is easily accessible, data shows that many online educational materials pertaining to surgical subspecialties are far above the average reading level in the United States. The aim of this study was to evaluate the English and Spanish online materials for the deep inferior epigastric perforator (DIEP) flap reconstruction procedure.

Methods

The first eight institutional or organizational websites that provided information on the DIEP procedure in English and Spanish were included. Each website was evaluated using the Patient Education and Materials Assessment Tool (PEMAT), Cultural Sensitivity Assessment Tool (CSAT), and either Simplified Measure of Gobbledygook (SMOG) for English websites or Spanish Orthographic Length (SOL) for Spanish websites.

Results

The English websites had a statistically lower CSAT score compared to the Spanish websites (p=0.006). However, Spanish websites had a statistically higher percentage of complex words compared to English sources (p<0.001). An analysis of reading grade levels through SMOG and SOL scores revealed that Spanish websites had statistically lower scores (p<0.001). There were no statistically significant differences in the understandability or actionability scores between the English and Spanish websites.

Conclusions

Online educational materials on the DIEP flap reconstruction procedure should be readable, understandable, actionable, and culturally sensitive. Our analysis revealed that improvements can be made in understandability and actionability on these websites. Plastic surgeons should be aware of what constitutes a great online educational resource and what online educational materials their patients will have access to.

## Introduction

The use of the internet to answer questions about health has increased over time. Currently, around one in four people are online worldwide, and 56% to 79% of internet users in the United States go online to obtain health information [1]. While the internet provides various opportunities for patients, such as finding clinicians, booking appointments, searching for medical advice, and understanding health conditions, there comes a great risk of health misinformation [1,2]. Along with this, many online health sources have the potential to be confusing if medical terms are unfamiliar to readers. The National Institutes of Health (NIH) and the American Medical Association (AMA) recommend that all health information be written at a United States sixth-grade reading level or below, a recommendation that followed the discovery that the average adult in the United States has a sixth to eighth-grade reading level [3,4]. This discovery is alarming, as lower health literacy is positively correlated with an increased cost of health care, increased morbidity, and non-adherence to medical treatment [5]. If patients are unable to read and understand health literature above their reading level, they will have worse health outcomes [5].

One study assessed the readability of online materials for patients going abroad for cosmetic procedures and found that the majority of materials were unregulated, lacking relevant information, and distributed by commercial providers [6]. Another study found that the readability of patient education materials for different surgical subspecialties ranges from a 10th-grade to college reading level, with educational material listed on the American Academy of Facial Plastic and Reconstructive Surgery website having a 12th-grade level readability [4]. It is imperative that individuals looking to undergo surgical or nonsurgical procedures have a good understanding of the procedure and can assess its risks and benefits, making the readability of online materials an incredibly important factor.

Individuals who are seeking an autologous breast reconstruction procedure will likely come across the deep inferior epigastric perforator (DIEP) flap reconstruction technique. In this procedure, tissue from the lower abdomen is used to recreate a breast mound [7]. Many pre-operative factors determine whether a patient would be a great candidate for this particular procedure, such as weight and prior abdominal surgery [7]. It is equally important that individuals looking to undergo DIEP flap reconstruction fully comprehend the post-operative risks and expectations of this procedure. Significant abdominal scarring, a post-operative hospital stay to monitor outcomes, and strict care instructions are all factors that patients should understand prior to receiving this procedure [7]. Although this responsibility falls primarily on the plastic surgeon, many plastic surgeons are limited in time available to meet and pre-operatively counsel patients. Therefore, many patients will inevitably use online resources to better understand their procedure and its risks.

The largest minority group in the United States is the Hispanic population, and Spanish is the most common non-English language spoken in U.S. homes [8]. Since such a large portion of the U.S. speaks little to no English, health information needs to be both accessible and readable by this population. However, one study performing a search of several common health conditions found that only 12% of Spanish links provided on the first page of the search led to relevant content for patients [9]. Additionally, the coverage of key information on Spanish health websites was inconsistent and poor [9]. To help patients make informed decisions about their healthcare, online materials about DIEP flaps should be accessible and readable by all individuals wanting this procedure, regardless of literacy level.

The purpose of our study is to assess the readability of the websites most visited for information on DIEP flaps that offer information in both English and Spanish. We aim to provide insight into whether the current online resources about DIEP flaps are readable based on well-established tools that determine the readability of educational materials.

## Materials and methods

Website/content selection 

Only academic, institutional, and organizational websites were utilized in this study. Websites were included if they were found within the first three hundred pages of Google and offered both English and Spanish information regarding DIEP flaps. The content was found with the search engine Google, utilizing the search term “DIEP flap surgery” in English and using the search term “cirugia de colgajo perforador de la arteria epigástrica inferior profunda” to find the Spanish counterparts. Any websites that did not have an English/Spanish counterpart, provided instructions to copy and paste the information into a translator tool, or any sponsored/advertised websites were excluded from this study. All content was accessed by a single investigator on April 11, 2023, utilizing an incognito search with all cookies, location, and user account information disabled. The eight websites with both English and Spanish versions were analyzed for their readability, cultural sensitivity, actionability, and understandability. 

Understandability and actionability analysis 

The Patient Education Materials Assessment Tool (PEMAT) was utilized to assess the understandability and actionability of the eight websites in both the English and Spanish versions [10]. This tool is a systematic and validated method to legitimize the literacy level of print and audiovisual patient education materials [11]. Understandability scores were gathered by answering nineteen questions on clarity of language, content selection, use of numbers, and level of organization. Actionability was assessed with eight questions regarding the use of explicit steps a patient can take, tools provided to aid in an action, and the quality of instructions given. The questions in each category were answered on a scale of “disagree (0),” “agree (1),” or “not available.” A percentage score is calculated for both the understandability and the actionability questions by adding the sum of points divided by the total number of questions answered (excluding questions answered not available). Websites with a higher percentage score are more understandable or actionable. 

Cultural sensitivity analysis 

Each website was assessed for its cultural sensitivity by evaluating the ability to respect and honor the different attitudes, beliefs, and behaviors of the intended audience [12]. The Cultural Sensitivity Assessment Tool (CSAT) was utilized to gather a cultural sensitivity score for each of the eight websites in both English and Spanish [13]. Each question was answered with a score of “strongly agree (4),” “agree (3),” “disagree (2),” “strongly disagree (1),” or “not applicable (0).” Thirty questions assessed the format, written message, and visual message. The cultural sensitivity score was determined by dividing the sum of the points by the total number of questions answered. A website was deemed culturally sensitive if the mean score was above 2.5.

Readability analysis 

The level of comprehensibility of each website (English and Spanish) was determined by the Readability Studio Professional Edition v2015.1 software (Oleander Software, Ltd, Vandalia, OH). The content of each website was downloaded into Microsoft Word 2021 (Microsoft Co, Redmond, WA) and stripped of all formatting, such as headings, bulleted items, and font, to ensure accurate analyses [14]. Each web link was run in the software to generate the readability analysis. The English websites were analyzed using the Simple Measure of Gobbledygook (SMOG). The Spanish materials were analyzed using the Spanish Orthographic Length (SOL) tool, a validated measurement of the SMOG tool in French, Spanish, and German [15]. The SMOG tool assesses readability through the use of words with 3+ syllables, calculation of grade reading level, function of word complexity, and sentence length [16].

Statistical analysis 

Bilingual evaluators were utilized to gather PEMAT and CSAT data from the eight Spanish and English sources. Each rater was instructed to answer the respective questions in their native language. To determine the agreeability between raters, a Krippendorff's alpha inter-rater analysis was utilized. Krippendorff's alpha values range from 0 (no agreeability, equal to chance) to +0.8 (strong agreeability). Values between 0 and 0.8 are interpreted as follows: 0.0-0.67 (low inter-rater reliability), 0.67-0.80 (acceptable inter-rater reliability), and 0.8+ (strong inter-rater reliability). Sample T-tests were performed to determine the level of differences between the eight Spanish and English sources. P values less than or equal to 0.05 were considered statistically significant.

## Results

After searching the first three hundred pages of Google, only eight websites fit the criteria to be included in this study. The eight English institutional/organizational websites that were utilized in this study were found in the first 124 search results (Table 1). About 62.5% of the Spanish websites included were found in the first 12 search results in Google (Table 2). Krippendorff’s Alpha Reliability estimate for the Spanish raters was 0.7219 and 0.7123 for the English raters, which both indicate acceptable inter-rater reliability.

**Table 1:** Ranking of websites evaluated.

Search result rank	Organization
1	A
8	B
28	C
51	D
59	E
60	F
113	G
124	H

**Table 2:** Spanish search term results.

Spanish search result rank	Organization
2	A
1	B
7	C
NA	D
4	E
NA	F
NA	G
12	H

Understandability and actionability analysis

Using the PEMAT analysis, the English websites had a mean understandability of 82.72%, an actionability of 53%, and an overall score of 67.86% (Table 3). The mean understandability, actionability, and overall score for the Spanish websites were 87.67%, 64%, and 75.84% respectively (Table 3). There was no statistically significant difference between the understandability scores of the English and Spanish sources (p=0.286) or the actionability score between the English and Spanish sources (p=0.352).

**Table 3:** PEMAT scoring of English and Spanish online materials. PEMAT: Patient Education and Materials Assessment Tool.

Institution/organization	Understandability (English)	Actionability (English)	Understandability (Spanish)	Actionability (Spanish)
A	78.9%	76%	85.8%	63%
B	90.7%	76%	91.7%	80%
C	91.4%	50%	95.8%	80%
D	85.8%	47%	95.8%	80%
E	86.4%	47%	91.7%	80%
F	64.9%	11%	78%	27%
G	88.6%	56%	90.8%	70%
H	75.1%	67%	71.7%	33%
Mean score	82.7%	53.8%	87.7%	64.1%

Cultural sensitivity analysis

The total Cultural Sensitivity Assessment Tool (CSAT) scores for the English and Spanish scores were 3.07 and 3.57, respectively (Table 4). Both scores are above the threshold (2.5) for the level of acceptability established by the CSAT creators. The English sources had a statistically lower CSAT score compared to the Spanish sources (p=0.006).

**Table 4:** CSAT scoring of English and Spanish materials. *One reviewer defined the website as non-acceptable by the CSAT standards. CSAT: Cultural Sensitivity Assessment Tool.

Institution/organization	Total CSAT score (English)	Interpretation (English)	Total CSAT score (Spanish)	Interpretation (Spanish)
A	3.11	Acceptable	3.57	Acceptable
B	3.13	Acceptable	3.78	Acceptable
C	3.49	Acceptable	3.74	Acceptable
D	3.19	Acceptable	3.85	Acceptable
E	3.43	Acceptable	3.82	Acceptable
F	2.66	Acceptable*	2.81	Acceptable*
G	3.13	Acceptable	3.58	Acceptable
H	2.38	Acceptable	3.4	Acceptable
Mean score	3.07	Acceptable	3.57	Acceptable

Readability analysis

The percentage of difficult words, defined as 3+ syllable words, was 15.24% in English and 24.20% in Spanish (Table 5). The Spanish sources on DIEP flaps had a statistically higher percentage of complex words utilized compared to the English sources (p<0.001). The average Simple Measure of Gobbledygook (SMOG) score indicating the reading level of each source was 11.73 in English (Table 5). The average Spanish Orthographic Length (SOL) score, the Spanish equivalent of SMOG, was 9.13 for the Spanish sources, which is significantly lower than the English material (p<0.001) (Table 5). English SMOG scores ranged from 10.05 to 13.02, while the Spanish SOL scores ranged from 7.77 to 10.46 (Table 5).

**Table 5:** Readability analysis. SMOG: Simple Measure of Gobbledygook; SOL: Spanish Orthographic Length.

Institution/organization	SMOG (English)		SOL (Spanish)	
	Reading grade level	SMOG hard words (%)	Reading grade level	SOL hard words (%)
A	12.49	13.53%	10.46	24.05%
B	11.94	19.47%	7.77	23.58%
C	11.21	10.72%	10	23.4%
D	10.05	11.96%	8.16	22.74%
E	10.29	11.92%	8.2	22.37%
F	13.02	19.74%	9.54	27.41%
G	12.42	14.92%	9.53	23.89%
H	12.43	19.64%	9.4	26.13%
Mean score	11.73	15.24%	9.13	24.2%

**Figure 1:**
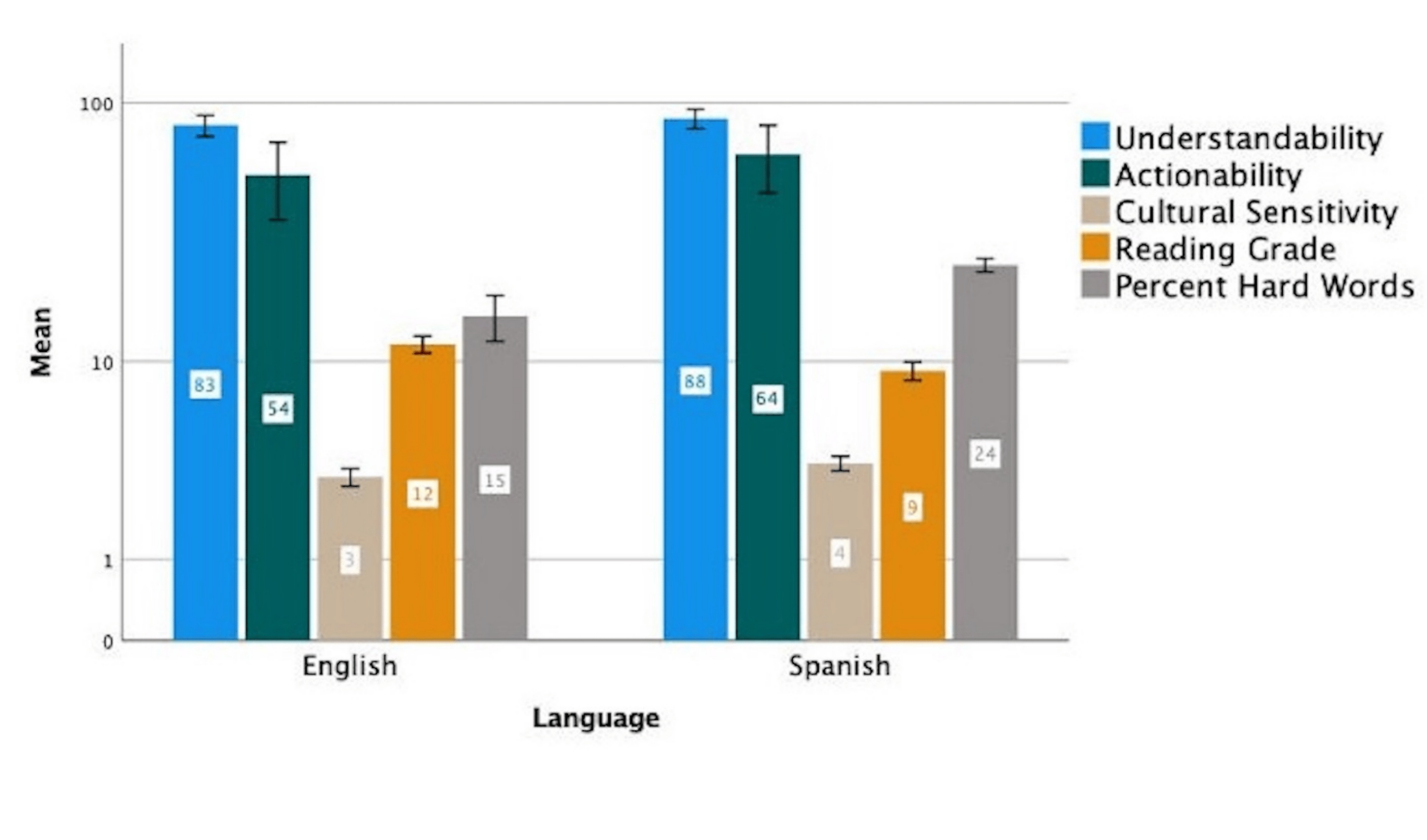
Results summary for English and Spanish online sources (95% CI).

For easier readability, all results are summarized in Figure 1.

## Discussion

To our knowledge, this is the first study analyzing the readability of English and Spanish online materials for the DIEP flap surgery. Two of the websites analyzed (organizations A and B) appeared within the top 10 searches in both languages. In the event a bilingual patient first searches for DIEP flap surgery information in English, there is likely a Spanish version of the website. Overall, the online materials assessed had good understandability, with a mean understandability score of 82.72% in English and a score of 87.67% in Spanish. The English sources had an understandability score ranging from 64.85% to 91.41%, while the Spanish sources had an understandability score ranging from 71.67% to 95.84%. Therefore, the heterogeneity of the website quality is similar in both languages, and there is no statistically significant difference in understandability. The main contributors to poor understandability scores included excess medical terminology and a lack of visual cues and images that support the message of the article. Complex medical jargon can lead patients to feel confused and isolated, affecting their health outcomes [17]. Lack of visual cues within educational materials can also impact patient understanding and health outcomes, with one study showing a statistically significant difference in health outcomes for individuals with low literacy levels when visual aids were utilized [18]. Additionally, no website provided a summary at the end for readers to review. These factors inevitably can cause patients to feel confused, making them less able to make the best healthcare choices for themselves.

Although patient understandability can be improved, the actionability score for each website was more concerning. Poor actionability scores were due to the absence of explicit steps outlined for the patient and tangible tools to aid in action for the patient. Only half of the websites identify one action the user can take, while none explain how to use the graphs, charts, diagrams, or tables to take action. This led to lower actionability scores for both languages, with English sources having a mean score of 53% and Spanish sources having a mean source of 64%. Actionability is important because it allows the reader to identify the next steps in care management, allowing patients to take initiative after learning new information. None of the websites provided information on how to locate a surgeon suited best for the patient’s needs, how to contact a plastic and reconstructive surgeon, what questions the patient should consider asking at their first appointment, or what steps should be taken prior to surgery. By leaving out this information, patients may become unnecessarily confused, leading to delayed operations for those who are good candidates for this procedure. A lack of information not only impacts the patients’ abilities to make informed decisions, but it interferes with patient autonomy. If an individual does not fully understand the surgery that they intend to have, they are agreeing to this procedure without proper education and acknowledgment of the associated risks.

All English and Spanish websites reviewed were considered acceptable by the CSAT scoring. The average English CSAT score was 3.07, which is statistically lower than the average Spanish CSAT of 3.57 (p=0.006). One of the main factors that led to higher scores for Spanish websites was the culturally neutral images found within the websites. Most images aimed to visually explain the procedure by only showing a person’s thorax as opposed to their thorax and head. This eliminated the possibility of cultural differences in hair colors, hair textures, and hair styles, but accurately depicted the physical features involved with the DIEP procedure, such as the breasts and abdominal region. Therefore, these websites were successfully able to create graphics that only show the physical features that would be common among the intended audience. However, none of the English or Spanish websites showed any graphics of this surgery on black individuals. This could inadvertently cause black patients looking to have this surgery to feel isolated, as they will have a harder time finding online resources with their skin tone. Furthermore, it is still concerning that the English websites underperformed on the CSAT, as nearly 241 million people in the U.S. speak only English [8]. Within the U.S., there are different cultural beliefs and ideologies, many of which vary by region. To not have culturally sensitive material readily available to patients is doing them a great disservice. Patients need to feel comfortable and represented when reading online health resources so they can have the best possible understanding of DIEP surgery. This will ultimately allow patients to make the best healthcare decisions for themselves.

The English and Spanish websites had statistically different complexity (reading grade level p<0.001; % hard words p<0.001), with English websites having both a higher reading level and Spanish websites having a higher percentage of difficult words. English and Spanish websites had an average reading level of 11.73 and 9.13 and an average percentage of difficult words of 15.24% and 24.2%, respectively. Both are significantly above the NIH and AMA recommendations for all written health information to be at or below a sixth-grade reading level [3,4]. While the average individual living in the U.S. reads at a seventh to eighth-grade level, one study found that the average reading score among fourth-, eighth-, and 12th graders was higher among White individuals compared to Hispanic individuals [19,20]. Additionally, as of 2022, only 20.9% of the Hispanic population aged 25 and older have a bachelor’s degree [21]. This percentage is lower than the non-Hispanic White population (41.8%), Black population (27.6%), and Asian population (59.3%) [21]. This means that even though Spanish websites have a significantly lower word complexity, the average Spanish-speaking minority patient will start off with a lower reading level. Therefore, word complexity and the percentage of difficult words still need to be lowered to adjust for potential barriers to health literacy, such as educational differences.

Spanish websites had a statistically higher percentage of hard words compared to English websites. On average, Spanish websites contained more words with 3+ syllables and used longer sentences to describe the DIEP surgery. The complexity of the Spanish websites is concerning, as patients with Spanish-language preferences are already considered to be part of a vulnerable group with less healthcare access [22]. Therefore, it is even more important for the online resources geared towards this community to be readable. If a patient is turning to online materials to independently research the DIEP surgery, they will likely encounter materials written at a high reading grade level with many complex words and medical jargon. Advising patients that material may be difficult to understand in advance will prevent them from becoming confused and overwhelmed. This will also provide an opportunity for the surgeon to strengthen the physician-patient relationship by inviting their patients to ask questions about their procedure when they cannot resort to websites for clarity. It is imperative that materials are at a level that can be understood well by the individuals undergoing this procedure because lower literacy rates are directly associated with a higher rate of poor health outcomes and mortality [23].

Study limitations

Patients seeking information about DIEP flaps may encounter various websites depending on their search engine history, which is placed into an algorithm to recommend materials specific to the patient. While the Google search engine was used for this study, patients may opt to utilize another search engine, such as Yahoo or DuckDuckGo, which could potentially provide different search results. Therefore, we cannot account for potential variability due to these causes, and further studies should be conducted to assess this.

Recommendations

Plastic surgeons should consider several factors when reviewing current online educational materials on DIEP flap surgery to better understand if those resources are appropriate for their patients. Materials should not only factor in readability, but also understandability, actionability, and cultural sensitivity. The information about DIEP flaps on the website for organization B is a good example of a well-rounded piece of educational material, scoring well on the PEMAT, CSAT, and readability analysis in both English and Spanish when compared to the other websites reviewed. Overall, our study found all English and Spanish websites explaining the DIEP flap surgery to be far above the recommended reading level. A study assessing the readability, suitability, and complexity of lower extremity reconstruction resources had similar results, with all online health information being near a 12th-grade reading level [24]. By being conscious of what websites patients are likely to encounter, plastic surgeons are better able to counsel patients on what to expect and make them cognizant of the potential complexity in advance.

## Conclusions

In conclusion, many of the top educational websites with articles on DIEP flap surgery were analyzed, revealing several gaps in understandability and actionability. By knowing what makes a good online resource, plastic surgeons can counsel their patients on what to expect when they are searching online for information pertaining to their surgery. Future studies should include a Spanish sub-group cultural analysis to better determine the unique linguistic needs and beliefs of each population. It is imperative for providers to recognize barriers patients may face in understanding a procedure and identify factors that make online resources culturally sensitive, readable, understandable, and actionable. Having a proactive approach to patient care by viewing the DIEP surgery through the eyes of the patient will allow plastic surgeons to create empowered patients who are able to make informed decisions about their health.
